# RNA-Targeting CRISPR–Cas Systems and Their Applications

**DOI:** 10.3390/ijms21031122

**Published:** 2020-02-07

**Authors:** Michal Burmistrz, Kamil Krakowski, Agata Krawczyk-Balska

**Affiliations:** Department of Molecular Microbiology, Biological and Chemical Research Centre, Faculty of Biology, University of Warsaw, 02-089 Warsaw, Poland; k.krakowski@student.uw.edu.pl (K.K.); akra@biol.uw.edu.pl (A.K.-B.)

**Keywords:** CRISPR–Cas, RNA, Cas9, Cas13, Cmr, Csm

## Abstract

Clustered Regularly Interspaced Short Palindromic Repeats (CRISPR)–CRISPR-associated (Cas) systems have revolutionized modern molecular biology. Numerous types of these systems have been discovered to date. Many CRISPR–Cas systems have been used as a backbone for the development of potent research tools, with Cas9 being the most widespread. While most of the utilized systems are DNA-targeting, recently more and more attention is being gained by those that target RNA. Their ability to specifically recognize a given RNA sequence in an easily programmable way makes them ideal candidates for developing new research tools. In this review we summarize current knowledge on CRISPR–Cas systems which have been shown to target RNA molecules, that is type III (Csm/Cmr), type VI (Cas13), and type II (Cas9). We also present a list of available technologies based on these systems.

## 1. Introduction

Clustered Regularly Interspaced Short Palindromic Repeats (CRISPR)–CRISPR-associated (Cas) (CRISPR–Cas) systems originate from Prokaryotes, where they serve primarily as a defensive mechanism against mobile genetic elements like phages and plasmids [1]. These systems consist of two components. The first is a genomic locus called CRISPR. It contains a series of short sequences of foreign origin called spacers that enable recognition of specific mobile genetic elements (MGEs) that were previously encountered [2,3]. Spacers are separated by repetitive regulatory sequences named repeats. Together with the leader sequence these spacers and repeats constitute the CRISRP array [4,5]. The second component of CRISPR–Cas systems are Cas proteins. These are coded by *cas* genes, usually located in the proximity of a CRISPR array. Cas proteins play an effector role in CRISPR–Cas systems.

The mechanism of CRISPR–Cas systems consists of three phases: adaptation, maturation, and interference. During the adaptation phase, new spacers are incorporated into the CRISPR array into its leader end [6]. During the maturation phase the CRISPR array is transcribed. The resulting transcript called pre-CRISPR RNA (pre-crRNA) is further processed into a set of CRISPR RNA (crRNA) molecules, each containing a single spacer flanked by fragments of a repeat sequence [7,8,9,10,11,12]. Subsequently, crRNAs are incorporated into ribonucleoprotein (RNP) complexes together with Cas proteins. RNP complexes scan nucleic acids searching for a sequence complementary to that encoded by crRNA [13]. Recognition of such a sequence triggers the interference phase that leads to degradation of a recognized nucleic acid [14,15,16]. The detailed mechanism of various types of CRISPR–Cas system has been reviewed elsewhere [17,18,19,20].

CRISPR–Cas systems are very common in the prokaryotic world, as they are detected in around half of the bacterial and archeal species known to date [17,21]. Numerous types have been described, which differ in many aspects like composition of Cas–crRNA complexes or structure of crRNAs. Current classification distinguishes two classes based on the structure of the effector complex [22]. Class 1 RNPs contain multiple subunits. On the contrary, class 2 systems utilize single subunit RNP complexes. Each class is further divided into types and subtypes, based on the presence of a specific Cas protein and the structure of the CRISPR loci.

Discovery of the CRISPR–Cas systems has revolutionized modern molecular biology. Due to their unique mechanism, they possess several features highly desirable for potential tools in this field. First of all, they are highly specific in terms of recognized sequence. Furthermore, this specificity can be easily altered by modifying the sequence coding the crRNA. The modular structure of the CRISPR locus makes these modifications even easier. The range of applications for CRISPR–Cas systems can be further expanded by modifying the Cas proteins themselves [23]. Most of the CRISPR–Cas systems described to date are those targeting DNA sequences. Among them, the CRISPR–Cas9 has proven to be the most convenient backbone for the development of new tools for research and biotechnology [24,25,26,27]. One reason for this is its simplicity, as it requires only one Cas protein for activity. In recent years several new types of CRISPR–Cas systems targeting RNA were discovered, paving the way for the development of new tools for research and biotechnology.

## 2. Overview of RNA-Targeting CRISPR–Cas Systems

### 2.1. Type III (Cmr/Csm) Systems

The type III systems are class 1 CRISPR–Cas systems. They are divided into four subtypes: III-A (Csm), III-B (Cmr), III-C, and III-D. Types III-A and III-B were first to be discovered, while types III-C and III-D were distinguished quite recently. The common feature of the type III systems is the presence of a Cas10, which usually contains two domains: the HD-type nuclease domain, and the palm domain [22]. Exceptions are type III-C (palm domain lacking cyclase activity), and type III-D (lack of HD domain) [28]. The typical RNP complex of this type consists of two parallel filaments. The first one is formed by six subunits of Cas7, and the latter by three subunits of a Cas11 homolog (Csm2 or Cmr5). The crRNA molecule is stretched along these filaments. The 5′ end of the crRNA, where the repeat derived handle is located, is capped by the Cas5 (Csm4 or Csm3) and Cas10 (Csm1 or Cmr2) proteins [29,30].

crRNA maturation in the majority of type III systems consist of two steps. The first is the digestion of a pre-crRNA transcript into units containing a single spacer sequence [7]. This stage is performed by Cas6. Interestingly, some type III systems can utilize Cas6 variant of other CRISPR–Cas types present in the cell to process the pre-crRNA [31]. Subsequently to this cleavage, crRNAs undergo secondary processing. The exact mechanism of secondary processing is not known. However, it was shown that this trimming generates two populations of crRNA molecules, differing in length [10,11].

**Figure 1:**
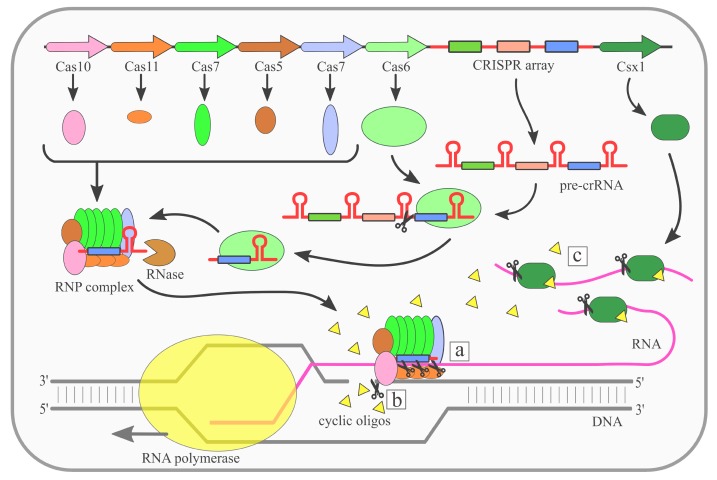
Mechanism of the Clustered Regularly Interspaced Short Palindromic Repeats (CRISPR)–CRISPR-associated (Cas) type III (Csm/Cmr) system. Cas6 endoribonuclease cleaves pre-CRISPR RNA (pre-crRNA) within the repeat region. Subsequently, the ribonucleoprotein (RNP) complex is assembled, while the 3′ end of crRNA is trimmed by an unknown nuclease. There are three nuclease activities of the RNP complex: (**a**) specific RNA cleavage, (**b**) non-specific ssDNA cleavage, (**c**) non-specific RNA degradation (details in the main text).

A unique feature of type III CRISPR–Cas systems is that they are the only known systems that use three nuclease activities (Figure 1). The first one is a sequence specific RNA cleavage performed by the Cas7 [30,32] (Figure 1a). The targeted RNA is positioned along the crRNA in the RNP and digested by the multiple copies of Cas7 present in the complex. As a result the RNA molecule is cleaved at fixed, 6-nt long intervals [33]. This so called ‘ruler’ mechanism is common for all type III systems [29,34,35,36]. It was shown that complementarity between crRNA and targeted RNA does not have to be strict, and the presence of mismatches does not abolish this nuclease activity [37]. The self-targeting of the transcripts that originate from the host’s own CRISPR array is avoided as it is transcribed in one direction only, and no transcripts complementary to crRNAs are generated.

The second nuclease activity of type III systems is non-specific ssDNA cleavage [36] (Figure 1b). This activity depends on the HD domain of Cas10, a metal-dependent DNase, and requires transcription of the protospacer sequence. The RNA polymerase opens the DNA double helix to start transcription exposing the antisense making it accessible for the HD domain of Cas10 of the RNP complex [38]. This metal-dependent DNase is activated by complementarity between crRNA and targeted RNA. The safety switch for this activity relies on complementarity between the 5′crRNA handle and the 3′ protospacer region of targeted RNA. Such complementarity inhibits cleavage, and thus prevents the host’s CRISPR array from being targeted [38]. The presence of the RNP complex in the close vicinity of the RNA polymerase is expected to be the result of the RNP complex scanning newly synthesized RNA.

Type III CRISPR–Cas systems present also a third nuclease activity, non-specific RNA degradation (Figure 1c). Similarly to non-specific ssDNA cleavage, this activity also depends on Cas10. However, in this case the main role is played by the palm domain. This domain is involved in the conversion of ATP into cyclic oligoadenylates [39,40]. Interestingly, this conversion is not constitutive. Similar to ssDNA cleavage, the non-specific RNA degradation is triggered by the binding of the RNP complex with targeted RNA with simultaneous non-complementarity between crRNA handle and targeted RNA [39,40]. Cyclic oligoadenylates allosterically activate the Csm6 or Csx1 proteins. These proteins are often present in type III CRISPR–Cas systems, but they are not incorporated into RNP complexes [41,42]. Csm6/Csx1 proteins contain two domains: the higher eukaryotes and prokaryotes nucleotide binding (HEPN) located at the C terminus, and the N-terminal CRISPR-associated Rossman fold (CARF) [43,44]. The CARF domain is believed to detect cyclic oligoadenylates produced by Cas10, while the HEPN domain is responsible for RNA cleavage.

The multiple nuclease activities of type III CRISPR–Cas systems raise questions concerning the reasons for maintaining such an extensive machinery to provide immunity against MGEs, while it could be achieved by CRISPR–Cas types with simpler structure. One of the postulated explanations is that type III systems enable bacteria to distinguish between lytic and lysogenic phage infection. Tolerating lysogenic infections while still being able to neutralize lytic infections would provide immunity without blocking the influx of potentially beneficial genes via horizontal gene transfer [45].

Another justification for the multiple nucleolytic activity of type III could be prevention of escape mutants avoiding recognition. Although DNase activity is sufficient to provide immunity against encountered MGE while the full crRNA/targeted RNA complementary is maintained, it is susceptible to mutations of the protospacer sequence [46]. This drawback is being covered by the RNase activity of Csm6/Csx1, which has been shown to be more flexible in tolerating mutations within protospacer sequences. This prevents the accumulation of foreign RNA, while the DNase activity adapts to the altered protospacer.

The third role of type III systems proposed by some authors is that they act as a suicide mechanism when a bacterial cell is being overrun by foreign nucleic acids [28]. However, no clear evidence has been provided so far that would confirm this hypothesis.

### 2.2. Type VI (Cas13) Systems.

**Figure 2:**
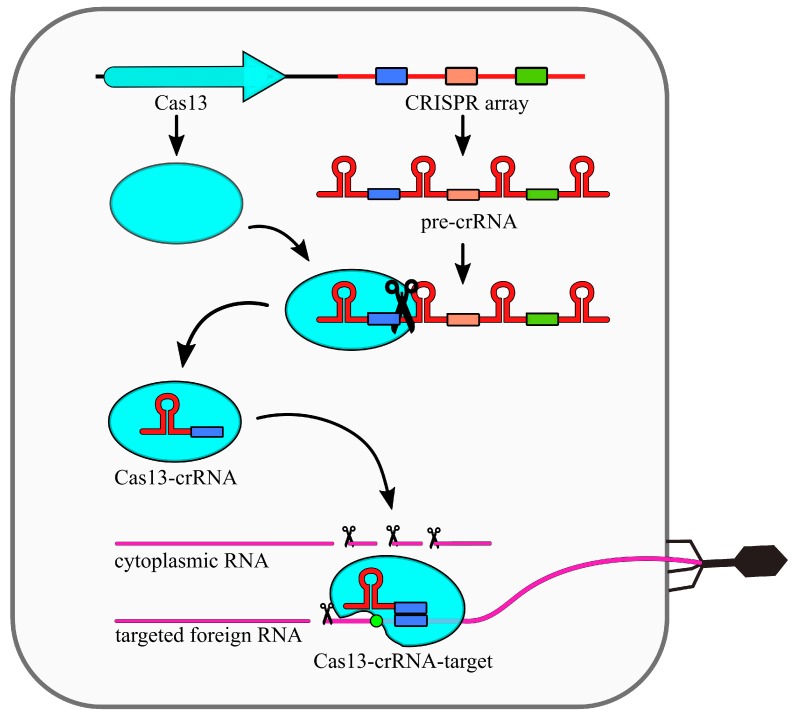
Mechanism of the CRISPR–Cas13 (type VI) system. The CRISPR array is transcribed into a long pre-crRNA transcript, which is subsequently processed into mature crRNAs by Cas13 protein. The crRNA-Cas13 complex scans the ssRNA searching for protospacer. Complementarity between crRNA and the protospacer sequence together with the presence of Protospacer Flanking Sequence (PFS) (green circle) induces conformational changes of Cas13, which results in higher eukaryotes and prokaryotes nucleotide binding (HEPN) domains activation and their displacement to the protein surface. This results in nonspecific RNA cleavage.

The type VI CRISPR–Cas systems present a relatively simple structure, as they require only one Cas13 protein and crRNA molecule for activity (Figure 2). To date, four subtypes have been distinguished: VI-A (that uses Cas13a variant, alternatively known as C2c2), VI-B (Cas13b/C2c6), VI-C (Cas13c/C2c7), and VI-D (Cas13d) [47,48]. Although the Cas13 of these subtypes differ in size and sequence, they all share a common feature, which is the presence of two HEPN domains [48,49]. These domains are responsible for RNA-targeted nucleolytic activity. HEPN domains are usually located close to different terminal ends of the Cas13 protein [50].

In general, processing of pre-crRNA into crRNA is performed by Cas13 itself in a metal-independent manner (with the exception of type VI-D) without the help of other host factors [48]. In subtypes VI-A, VI-C, and VI-D crRNAs contain a repeat-derived handle on their 5′ end. On the contrary, subtype VI-B generates crRNAs with handle on the 3′ end [49]. Secondary processing is presumably performed by other host nucleases. It was shown that crRNA maturation is not necessary for type VI activity, and even unprocessed pre-crRNA is sufficient for recognition of targeted RNA [51]. The complex of Cas13 and cRNA presents no nucleolytic activity until it binds to targeted ssRNA. Binding between crRNA and targeted RNA triggers the conformation change of the RNP complex. Both HEPN domains are moved closer to each other, creating a single catalytic site [52]. As this site is located at some distance to crRNA-targeted RNA duplex, it is expected to cleave not only the targeted RNA but also any other ssRNA, including the host’s own RNA, in close proximity of a RNP complex. This is sometimes referred to as ‘collateral damage’ or ‘collateral cleavage’ [47,49,53]. Some authors speculate that the reason for this sequence-specific activated unspecific RNA cleavage is to cope with bacteriophage infection either by cleaning the cell of the all RNA and thus impeding phage replication, or by inducing cell dormancy/death in order to protect neighboring cells [54].

Similar to other CRISPR–Cas types, the type VI systems utilize a safety-lock mechanism that prevents from activating the system by the host’s own RNA. Apart from subtype VI-D [53], and some examples of VI-A [55], all type VI systems require a so called Protospacer Flanking Sequence (PFS) located in the direct vicinity of a protospacer sequence. This sequence varies between different subtypes. For example: LshCas13a uses a non-G PFS located at 3′ end [47], whereas BzCas13b requires the double-sided PFS of non-C upstream of the target site and NAN or NNA downstream of the target site [49]. The nucleolytic activity of some Cas13 variants can be modulated by other proteins. For example, two proteins: Csx27 (repressor), and Csx28 (stimulator) have been shown to regulate Cas13b activity [49].

### 2.3. Type II (Cas9) Systems

The type II CRISPR–Cas systems belong to class 2 and is divided into three subtypes (II-A, II-B, and II-C). In addition to CRISPR array and Cas proteins this type encodes additional trans activating RNA (tracrRNA), which mediates interaction between crRNAs and Cas9.

The primary processing of type II pre-crRNAs is performed by RNase III [9]. The 5′ fragment of tracrRNA binds to a repeat sequence, creating a duplex which is cleaved by RNase III [8]. Subsequently, the 5′ handle of this intermediate crRNA is further trimmed by an unknown nuclease. The maturated crRNA/tracrRNA duplex is incorporated into Cas9.

**Figure 3:**
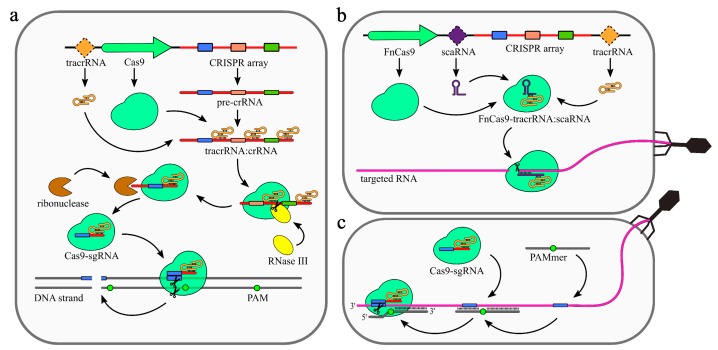
Mechanism of the CRISPR–Cas9 (type II) system. (**a**) DNA targeting CRISPR array transcription generates pre-crRNA. Maturation of the crRNAs is dependent on trans activating RNA (tracrRNA), which is partially complementary to the repeat sequences in the pre-crRNA resulting in tracrRNA/crRNA duplex formation. Those duplexes are bound and stabilized by Cas9 protein. Host RNase III is then recruited to cleave pre-crRNA into units containing single spacer sequences. Further trimming of the crRNAs is performed by unknown ribonuclease. The complex of Cas9 and single guide RNA (sgRNA: tracrRNA–crRNA) scans DNA until it finds a Protospacer-Associated Motif (PAM) sequence. The DNA strand is then unwound, allowing sgRNA for complementarity verification. Positive recognition results in cleavage of both DNA strands. (**b**) scaRNA-dependent RNA targeting was observed for Cas9 from *Francisella novicida* (FnCas9). Small CRISPR/Cas-associated RNA (scaRNA) hybridizes with tracrRNA to form heteroduplex that binds Cas9 protein. FnCas9–tracrRNA/scaRNA complex targets RNA partially complementary to scaRNA sequence. The precise mechanism of FnCas9 remains unclear. (**c**) PAM-presenting oligonucleotide (PAMer)-dependent RNA targeting Functional Cas9–sgRNA complex is able to target RNA in the presence of PAMmers–short DNA oligonucleotides containing PAM. When the PAMmer is bound to target RNA, the Cas9–sgRNA complex is able to recognize and cleave the RNA as long as complementarity between sgRNA and targeted RNA is maintained.

Similar to type III systems, Cas9 was shown to possess several nucleolytic activities (Figure 3). The most common nuclease activity among type II systems is specific dsDNA cleavage (Figure 3a). Upon binding with crRNA/tracrRNA duplex, Cas9 undergoes a conformational change that enables it to bind and scan DNA [56]. Recognition begins with identification of the Protospacer-Associated Motif (PAM) located at the 5′ end of the protospacer sequence. Following that, base pairing between crRNA and targeted DNA takes place, which activates the DNase activity of Cas9. The strand containing complementary to crRNA and the displaced strand are cleaved by HNH and RuvC domains, respectively [9,56].

For some type II CRISPR–Cas systems, additional nucleolytic activities have been described. Unlike the hallmark DNase activity of type II, these are directed against RNA, rather than DNA. An example of such activity is the type II system of *Francisella novicida* [57] (Figure 3b). Apart from elements of a typical type II system, the genome of *F. novicida* encodes a small, CRISPR–Cas-associated RNA (scaRNA) 5′ fragment which is complementary to the tracrRNA and 3′ shows similarity to the gene encoding a bacterial lipoprotein. It was shown that this scaRNA is responsible for downregulation of the lipoprotein transcript in a mRNA targeting mechanism involving scaRNA, tracrRNA, and Cas9. Moreover, neither the CRISPR array, nor the HNH or RuvC domains, are necessary for this activity. It is supposed that scaRNA replaces the crRNA in the RNP complex. However, the exact mechanism has not been described to date.

Recent studies have shown that some of the type II CRISPR–Cas systems present natural nucleolytic activity against ssRNA targets. What differs this activity from the one described for *F. novicida* is that it does not utilize scaRNAs. It was shown that this activity is crRNA-, and tracrRNA-dependent, and that a site specific RNA cleavage is most probably performed by the Cas9′s HNH domain [58]. Neither PAM nor PFS sequences were shown to be required to regulate this activity [59]. To date, this particular ssRNA targeting activity has been described for *Staphylococcus aureus* [60], *Campylobacter jejuni* [58], and *Neisseria meningitides* [59]. The exact role of the ssRNA targeting activity of type II CRISPR–Cas systems remains unknown.

## 3. Applications Based on RNA-Targeting CRISPR–Cas Systems

As previously mentioned, the possible range of applications based on RNA-targeting is impressive. Table 1 lists the applications developed to date.

### 3.1. Type II(Cas9)-Based Applications

One of the successful attempts of reengineering the type II CRISPR–Cas system was performed for *Streptococcus pyogenes* [61]. The native Cas9 was redirected to target ssRNA in vitro by introducing short PAM-presenting oligonucleotide (PAMer) (Figure 3c). The presence of PAMer replaces to some extent the PAM-presenting non cleaved strand, which is required for Cas9 activation. The introduction of mismatches within a PAMer sequence prevents degradation of the PAM-less genomic sequences, while the RNA cleavage is maintained. Furthermore, the length of a given PAMer can be modified to modulate the specificity and efficiency of the ssRNA cleavage. In addition to the RNA targeting, the PAMer-based approach was applied for catalytically inactive Cas9 to provide programmable RNA-guided RNA tracking in live cells [65], as well as pulling down specific RNA [61]. As an alternative to using PAMers, RNA specific cleavage can be achieved by fusing inactivated Cas9 (dCas9) with exogenous nuclease. This is possible due to the fact that RNA binding by Cas9 is PAM-independent [65,74]. One such system was developed by fusing dCas9 with PIN RNase domain [74].

Many variants of Cas9 target RNA naturally, without need of a PAM sequence [57,58,59,60]. Some of them were also shown to be programmable by alteration of the spacer sequences [58,59]. These Cas9 variants look very promising candidates for potential applications. However, knowledge of their exact mode of action and regulatory factors is still limited. It has been reported that Cas9 RNA cleavage is affected by the presence of secondary structures in targeted RNA [60].

### 3.2. Type VI(Cas13)-Based Applications

Cas13 has become a backbone for multiple applications. It has been used to knock-down RNA in bacteria [47], plant cells [71], and several types of mammalian cells [53,55,66]. Cas13d from *Ruminococcus flavefaciens* was truncated to generate its minimal functional variant [75]. The reason for that was to obtain a Cas13 was small enough to be delivered by viral vectors. The collateral RNase activity of Cas13 was observed in bacterial studies, while researchers using eukaryotic cells did not report such activity.

The collateral RNA cleavage associated with Cas13 may be considered by some as a drawback. However, it may also prove itself beneficial in some applications. An example for that is its utilization as a backbone for in vitro detection method of DNA and RNA named Specific High-Sensitivity Enzymatic Reporter unLOCKing (SHERLOCK) [67]. In this method, the nucleic acid sample is amplified to dsDNA using recombinase polymerase amplification, which for RNA samples is combined with reverse transcription. The obtained dsDNA serves as a template for T7 polymerase, which generates ssRNA transcripts. These are subjected to Cas13 scanning. Upon recognition, the activated Cas13 cleaves reporter via collateral RNase activity, thus releasing the signal. The SHERLOCK method is highly specific and sensitive, and has been already successfully applied for detection of genomic fragments of Zika and dengue viruses [67].

The SHERLOCK method was further improved to allow multiplex detection (SHERLOCKv2) [68]. This was achieved by combining multiple variants of Cas13. Each variant prefers a different motif when cleaving RNA (AU, AC, UC, or GA). Thanks to that, different reporters can be utilized to detect various RNAs in one sample. To improve signal strength SHERLOCKv2 uses a Csm6 protein that is allosterically activated by Cas13 through cyclic oligoadenylates upon its own activation. The combined collateral RNA cleavage of Csm6 and Cas13 results in a stronger signal from cleaved reporters [68].

In addition to RNA knock-down, Cas13 was successfully used in RNA-tracing and RNA-editing. A catalytically inactive Cas13 (dCas13a) was developed and used in RNA immunoprecipitation and RNA-imaging [68]. Another inactivated Cas13 (dCas13d) was used in research concerning crRNA guided regulation of mRNA splicing [64]. Another example of technology using inactivated Cas13 is RNA Editing for Programmable A-to-I Replacement (REPAIRv1) [66]. In this system, the inactivated type VI Cas13 (dCas13b) was fused together with human adenosine deaminase acting on RNA (ADAR) enzymes, namely ADAR1 and ADAR2. This enabled programmable replacement of adenosine with inosine, which is recognized as a guanine during complementary base pairing. Thus, from the functional perspective this can be considered as an A to G point mutation within a selected site of mRNA. The A-to-I replacement allows for temporal alteration of translation of a selected gene. The specificity of this system was further improved by introducing a mutation within the ADAR2 sequence (REPAIRv2) [66].

### 3.3. Type III-Based Applications

The complexity of type III effector complexes makes their utilization challenging. Nevertheless, they have been successfully applied in studying the role of prokaryotic genes [62]. Active type III complexes were reconstituted in vitro [32,76,77,78]. The Csm6, which is a RNase harnessed by type III CRISPR–Cas systems, was used in the SHERLOCKv2 method of specific RNA detection [68].

## 4. Summary

Identification of the RNA-targeting activity of CRISPR–Cas systems types II (Cas9), III (Cmr/Csm), and VI (Cas13) has significantly broadened the range of potential applications for these systems. Tracking, targeting, or editing specific RNAs facilitates both research and development of new therapies. The advantages of focusing on RNA are clearly visible when researching genes for which deletion of is lethal, or when developing gene therapy. In the case of RNA-level modifications, the effects of these are temporary and are less likely to cause permanent changes in genetic material of the patient. It must be noted that RNA-targeting CRISPR–Cas systems are not perfect, and their utilization is facing challenges such as collateral cleavage, off target activity, troublesome delivery of PAMers, significant size of components limiting choice of delivery methods, or even possible immunogenicity that is caused by using proteins of bacterial origin in mammalian cells. The potential clinical applications of RNA-targeting CRISPR–Cas systems include but are not limited to: rapid detection of various nucleic acids [67,68], treatment of diseases related to G to A mutations using RNA editing [66], treatment of genetic diseases using RNA knockdown, treatment of diseases related to incorrect mRNA splicing and high-throughput drug discovery [79]. Certainly, present and future technologies based on RNA-targeting CRISPR–Cas systems will facilitate advancements in both research and therapeutic applications.

## Figures and Tables

**Table 1:** Applications based on RNA-targeting CRISPR–Cas systems.

Application	CRISPR–Cas Type (Method Name)	Key Features	Reference
RNA knockdown	Cas9	programmable and specific RNA cleavage targeted RNA sequence does not require PAM motif as it is delivered by PAMer DNA corresponding to targeted RNA sequence is not affected	[61]
	Cas13a/b/c	requires Cas13 variant and CRISPR array for activity multiple transcripts can be targeted simultaneously usually PFS is required in targeted RNA possible off target cleavage	[47,49,51,53,55]
	Csm/Cmr	utilizes three nuclease activities: specific RNA cleavage, non-specific ssDNA cleavage, non-specific RNA degradation complex structure of effector complex limits its application	[62,63]
	Cas13d (CasRx)	the smallest Cas13 variant known to date possible to be delivered by Adeno-Associated Virus (AAV) no PFS required good efficiency and specificity	[64]
RNA imaging and tracking	dCas9	dCas9 is fused to a fluorescent protein and nuclear localization signal enables recognition of endogenous, untagged, unmodified mRNA PAM is delivered by PAMers method does not disturb the functional mRNA and protein levels	[65]
	dCas13a	catalytically inactive Cas13 is fused to GFP, zinc finger and The Krüppel-associated box domain (KRAB) domain enables recognition of endogenous, untagged, unmodified mRNA negative feedback system based on self-targeting zinc finger and repression of KRAB domain reduces background signal from unbound protein	[55]
RNA editing	Cas13b (REPAIR)	dCas13b is fused to ADAR2 domain enables to convert selected adenosine to inosine small size enables delivery via AAV	[66]
nucleic acid detection	Cas13a (SHERLOCKv1)	requires Cas13a and quenched fluorescent RNA reporter specific recognition of the target triggers nonspecific RNA cleavage that activates the fluorophore rapid, inexpensive RNA detection single-base mismatch specificity attomolar sensitivity can be utilized in detection of specific strains of pathogens, genotyping, and identification of mutations	[67]
	Cas13 + Csm6 (SHERLOCKv2)	extended version of SHERLOCKv1 Csm6 strengthens the signal multiplex detection of up to four targets 3.5-fold increased sensitivity	[68]
splicing alteration	dCas13d (dCasRx)	catalytically inactive CasRx is fused with negative splice factor hnRNPa1 exon skipping can be achieved by targeting cis-acting motifs like: intronic branch point, splice acceptor site, exon, splice donor site	[64]
resistance against RNA viruses	FnCas9	PAM independent cytosol localization of FnCas9 is necessary to inhibit RNA viruses nuclear localization is needless, thus potential off-targets of FnCas9 are limited nuclease activity is not required production of RNA virus resistant plants possible multiplex targeting	[69,70]
	Cas13a	provides stable immunity to viruses in plants codon optimization of Cas13a is required for proper protein functioning possible off-target activity multiplex targeting possible	[71,72]
induction of apoptosis	Cas13a	programmed cell death is triggered by nonspecific RNA degradation in response to infection by cellular pathogen	[47]
regulation of gene expression	Cas13b	catalytically inactive Cas13 is fused with eukaryotic RNA-modifying enzyme N^6^-methyladenosine (m6A) variant fused to YTHDF1 protein can trigger assembly of translation machinery variant fused to YTHDF2 protein can induce RNA degradation	[73]
specific RNA isolation	dCas9	catalytically inactive Cas9 is fused with biotin suitable for purification of tagless RNA transcripts affinity chromatography purification of biotinylated dCas9-gRNA-targetRNA on streptavidin resin RNA sequence of interest does not require PAM motif as it is delivered by PAMer	[61]
elimination of repetitive sequences	Cas9	dCas9 is fused to the PIN RNA endonuclease no PAMer is required small size enables delivery via AAV potential application in treating genetic diseases connected with toxic microsatellite expansions	[74]

## Abbreviations

AAV	Adeno-Associated Virus
ADAR	adenosine deaminase acting on RNA
CARF	CRISPR-associated Rossman fold
Cas	CRISPR-associated (protein)
CRISPR	Clustered Regularly Interspaced Short Palindromic Repeats
crRNA	CRISPR RNA
dCas	catalytycally inactive Cas protein
HEPN	higher eukaryotes and prokaryotes nucleotide binding
KRAB	The Krüppel-associated box domain
MGEs	Mobile Genetic Elements
PAM	Protospacer-Associated Motif
PAMer	PAM-presenting oligonucleotide
PFS	Protospacer Flanking Sequence
REPAIR	RNA editing for programmable A-to-I replacement
RNP	ribonucleoprotein
scaRNA	small, CRISPR–Cas-associated RNA
sgRNA	single guide RNA
SHERLOCK	Specific High-Sensitivity Enzymatic Reporter unLOCKing
tracrRNA	Trans-activating crRNA
